# Web-Based Violence Risk Monitoring Tool in Psychoses: Pilot Study in Community Forensic Patients

**DOI:** 10.1080/15228932.2016.1128301

**Published:** 2016-01-29

**Authors:** Gautam Gulati, Robert Cornish, Hasanen Al-Taiar, Christopher Miller, Vivek Khosla, Christopher Hinds, Jonathan Price, John Geddes, Seena Fazel

**Affiliations:** ^a^ Oxford Health NHS Foundation Trust, Oxford, England; ^b^ Department of Psychiatry, University of Oxford, Oxford, England

**Keywords:** psychoses, risk assessment, Schizophrenia, technology, violence

## Abstract

We describe the development and pilot testing of a novel, web-based, violence risk monitoring instrument for use in community patients with psychoses. We describe the development of the tool, including drawing on systematic reviews of the field, how item content was operationalized, the development of a user interface, and its subsequent piloting. Sixty-eight patients were included from three English counties, who had been discharged from forensic psychiatric services. Over 12 months, 310 questionnaires were completed on the sample by professionals from several disciplines and qualitative feedback collected relating to the use of the tool using an electronic survey. Strengths of this approach for risk assessment, and potential limitations and areas for future research, are discussed.

Violence risk assessment is widely used in clinical practice in many countries (Singh, Desmarais, et al., 2014). Such assessment in a psychiatric setting may constitute an aspect of assessment for court or assist the monitoring of individuals in forensic psychiatric hospitals or in the community (Dunn et al., 2014). In the latter context they are used to make decisions about discharge from hospital (Davoren et al., 2013) and recall to hospital from the community.

Violence risk assessment in psychiatric practice has moved from unstructured assessments to the use of structured instruments that are completed at specific time points in the patient pathway. There has been debate about which type of structured instrument is preferable: structured clinical judgement tools with categorical outcomes based on the application of professional judgement to validated risk factors, or actuarial instruments with numerical outcomes (Hilton, Harris, & Rice, 2006). Clinicians, however, tend to prefer methods that identify risk factors and interventions rather than those with a numerical probability (Heilbrun et al., 2004). Structured clinical judgment tools are commonly used and although they may have advantages over actuarial measurements of violence risk, such as focusing attention on factors that may be treatable, there are considerable limitations in the predictive validity of both such approaches (Singh, Serper, Reinherth, & Fazel, 2011). In addition to low positive predictive values, instruments have limitations in specificity and a reliance on static factors (Fazel, Singh, Doll, & Grann, 2012). Their clinical utility is also undermined by differences in what would constitute a high-risk categorization (Singh, Fazel, Gueorguieva, & Buchanan, 2014). The risk assessment process in practice is time-consuming, with one study reporting that it takes 15–16 person hours on average to complete (Viljoen, McLachlan, & Vincent, 2010). Therefore, current practices in risk assessment do not mirror the time-sensitive and dynamic real-world nature of risk assessment that would be expected in forensic community-based mental health care.

In this study, we describe the development of a novel web-based instrument, and experience of piloting its use in a community forensic mental health service in the United Kingdom. This setting involves the care of individuals with a significant history of violence, and requires more dynamic risk monitoring than individuals treated in a general psychiatry setting. The majority of patients treated in such a setting would have a primary psychotic diagnosis with or without comorbid personality disorder and substance misuse. Similar services designed for mentally disordered offenders exist in other jurisdictions including the United States, Northern Europe, Australia, and Japan (Fuji, Fukuda, Ando, Kikuchi, & Okada, 2014; Hayes, Kemp, Large, & Niellsen, 2013; Pinals, 2014), but rates of patients per head of population vary widely (Priebe et al., 2008).

We sought to develop an instrument that was evidence-based, based on dynamic factors, user-friendly at the point of input, and that would not only assist with effective risk monitoring but also provide a visual graphic output to inform decision making in day-to-day clinical practice in a community forensic psychiatry setting.

The use of technology to monitor clinical parameters has already been shown to be feasible in mental health patients (Luxton, June, & Kinn, 2011; Miklowitz et al., 2012), as well as in other health care settings (Luxton, McCann, Bush, Mishkind, & Reger, 2011; Stanton, Willis, & Balanda, 2000). In particular, a similar interface that was developed for mood-related monitoring of patients with bipolar disorder (Miklowitz et al., 2012) showed translational benefits in terms of quality of care and prioritization of health care resources.

We sought to develop a novel instrument, rather than operationalize an existing empirically tested instrument such as the HCR-20, for several reasons. Firstly, no current widely used empirically tested tool has availability on an online platform. Secondly, we aimed to develop a tool with an emphasis on dynamic factors only, given that we intended to use this in a population with relatively high static factors, as would be expected in our community forensic service setting, which caters for discharges from a medium-security facility. Thirdly, we noted that the most up-to-date review of evidence (Witt, van Dorn, & Fazel, 2013) highlighted risk factors that are not included in currently used instruments such as the HCR-20. Lastly, we aimed to develop a tool that was quick to administer and needed little training.

The aim of this paper is primarily descriptive: seeking to present the methodology in the initial development of the instrument, and to outline user experience of piloting it over a 12-month period. The approach incorporates both a novel risk assessment tool and a way of collecting, recording, and sharing the data gathered from the risk assessment tool.

## Methods

### Stage 1: Designing the instrument

In developing the instrument (named Foxweb), the following steps were involved: A systematic review of the literature relating to risk factors for violence in individuals with psychoses was undertaken, which was published elsewhere (Witt, van Dorn, & Fazel, 2013). From the risk factors analyzed, we extracted 10 risk factors that were most statistically significant to include in a tool. There was agreement sought about the clinical relevance of these factors, done through a series of meetings between the study authors (GG and SF), and discussed with other study authors, where clinical and academic consensus was reached. We agreed on 10 factors to rationalize the ease and acceptability of the instrument. There was also consensus that additional factors would add little to the scope of the instrument in day-to-day community forensic practice. We selected dynamic factors primarily, as we intended to use the tool for patients discharged from a medium-security setting to mirror clinical practice, where the monitoring of such factors informs treatment interventions and decisions around recall to hospital.

Operationalization of risk factors was completed by examining the questionnaires and rating scales used in the primary studies that were included in the systematic review, and using the wording and guidelines in the validated tools that made up the primary studies. Where more than one rating scale was a possibility for any given risk factor, a clinical and academic consensus was reached as to the most appropriate scale to be used. For six factors, a Likert scale was used, with the remaining four variables rated dichotomously. A list of the risk factors agreed and the references for the tool used are presented in Table 1.

**Table 1.** Risk Factors Used in the Foxweb Risk Monitoring Instrument

Risk factor	Reference used for operationalizing definition
Increased aggression	Coccaro, Berman, & Kavoussi, 1997
Increased impulsivity	Kay, Fiszbein, & Opler, 1987
Emergence of increasing anger	Novaco, 1975
Drug misuse in the past week	Bartels, Drake, Wallach & Freeman, 1991
Alcohol misuse in the past week	Bartels, Drake, Wallach & Freeman, 1991
Emergence/exacerbation of paranoid/persecutory delusions or passivity	MacArthur-Maudsley Delusion Assessment Schedule; screening questions (Applebaum, Robbins, & Mohanan, 2000)
Emergence of nonadherence with treatment	Ellouze et al. 2009
Become homeless in the past week	Fazel, Khosla, Doll, & Geddes, 2008.
Violent victimization in the past week	MacArthur Community Violence Interview—as used in CATIE (Swanson et al., 2006)
Suicide attempt in the past week	Hawton, Zahl, & Weatherall, 2003

An operational manual for use with the Foxweb tool was developed and written at a level intended to be useable by any mental health care professional, with an emphasis on simplicity of use and clarity of definitions.

A secure web-based portal was designed in conjunction with a technology team at the university department, where the assessment of relevant risk factors could be entered on an electronic questionnaire. Time-based graphical output in the form of line graphs and “blobbograms” could be made available for any given time period for a given patient to health care professionals with a unique secure log-in.

The technical teams were based at the Department of Psychiatry, University of Oxford, and had UK disclosure and barring service clearances. The portal was designed with strict role-based access control, ensuring that users were able to see only the data required to fulfil their role. An audit record of every action taken within the system was retained. Information was held in a physically secure, access-controlled server room within an NHS hospital with high-level encryption measures.

### Stage 2: Pilot study

The pilot study investigated the ease of use and feasibility of the instrument. We obtained approval from the Community Services Clinical Governance Committee within the Specialised Services Division of Oxford Health Foundation Trust in May 2013 with a view to piloting the tool within the Forensic Community Mental Health Team caring for patients across three English counties (Oxfordshire, Buckinghamshire, and Berkshire) as a service evaluation.

Community patients with primary diagnoses of psychoses (schizophrenia, schizo-affective disorder, bipolar disorder, delusional disorder, psychotic depression, and drug-induced psychoses) who were in forensic psychiatric services were included. Patients with a primary diagnosis of psychoses were chosen, as these represented the vast majority of community patients in a forensic service, and as the instrument was based on risk factors extracted from a review specifically relating to psychoses (Witt, van Dorn, & Fazel, 2013).

**Figure 1.**
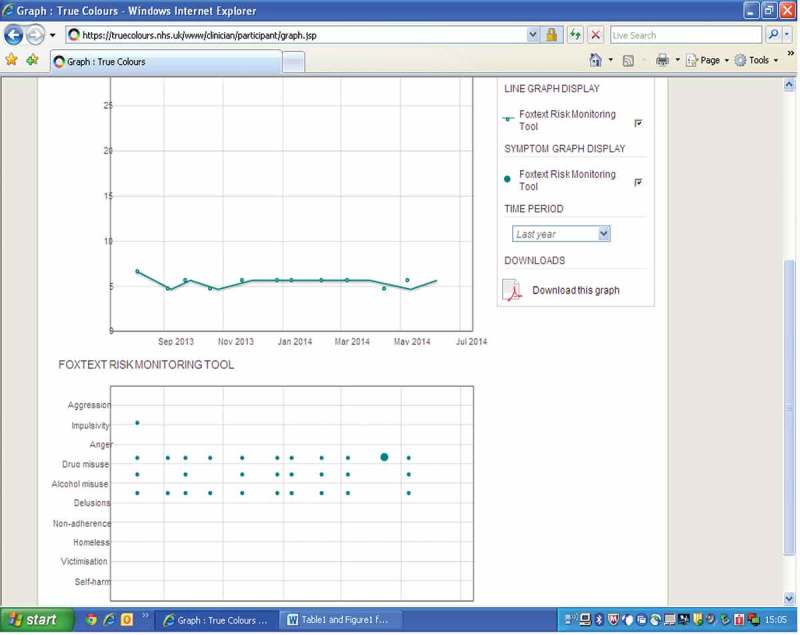
Example of graphical output from Foxweb.

Clinicians working within the Forensic Community Mental Health Teams were invited to enter risk-monitoring data on each occasion that a patient was seen. Graphical output was available to any member of the team, and weekly graphs were also e-mailed on request. Graphs were automatically uploaded monthly to the patient’s electronic health record so that they could be viewed in clinic without the need for a separate log-in. The output was in the form of line graphs and blobbograms showing the evolution of risk factors over time (see Figure 1 for an example).

### Step 3: Feedback on use of the instrument

Professionals using the instrument were surveyed at the end of the one-year period using Survey Monkey, an Internet-based survey tool. They were surveyed in relation to their experience of using the Foxweb instrument, which evaluated five questions, with four requiring a Likert-style response and one requiring a numerical response. The questions are listed in Table 2.

**Table 2.** Questions Used in Survey of Clinicians for Feedback in Relation to the Instrument

1	How relevant was the instrument to your day-to-day clinical practice?	1 = not at all satisfied
2	How useful was the instrument to your day-to-day clinical practice?	2 = slightly satisfied
3	How relevant did you feel were the risk factors included in the monitoring instrument?	3 = somewhat satisfied
4	How would you rate the ease of use of the instrument?	4 = very satisfied
5	How long, in minutes, did it take to complete individual questionnaires?	5 = extremely satisfiedRequired a numerical response

## Results

Over a period of 12 months, 310 risk questionnaires were completed for 68 patients. This included 63 male patients and 5 female patients with ages ranging 25–72 years (mean 44.1 years). Of the patient sample, 30 were undergoing voluntary treatment, 8 were on a treatment order or leave from civil detention, and 30 were under an order imposed by a court. Diagnoses included schizophrenia (*n* = 53), schizoaffective disorder (*n* = 8), bipolar disorder (*n* = 4), psychotic depression (*n* = 1), persistent delusional disorder (*n* = 1), and acute and transient psychosis (*n* = 1). Patients were predominantly White British (*n* = 40), African Caribbean (*n* = 6), or Asian (*n* = 6). For 16 patients, ethnic origin was not known.

Completed questionnaires resulted in data being added and outputs being displayed as both blobbograms and line graphs. These graphs were available to assessing clinicians to view prior to and after the addition of data. On the blobbogram, the size of the blob on the horizontal axis was proportional to the numerical score entered by the user. The pattern of evolution for a given risk factor was therefore visible, as the *x* axis of the blobbogram represented time. An additional line graph on the output screen charts total score (from all 10 items) on the *y* axis against time on the *x* axis, once again looking to demonstrate the evolution of risk factors over time (see Figure 1 for an example).

We found clinicians of different disciplines and across all three counties were willing to enter data. The occupations of those providing and accessing data included psychiatrists (*n* = 3), social workers (*n* = 4), an occupational therapist (*n* = 1), and community psychiatric nurses (*n* = 4).

**Figure 2.**
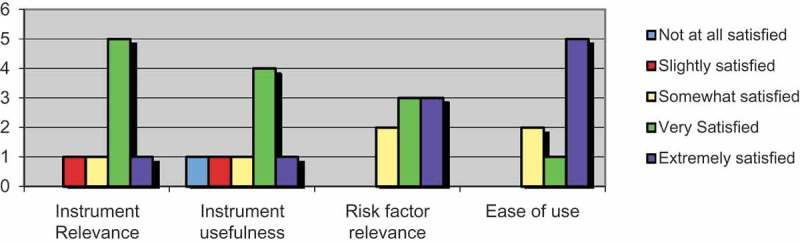
Responses from user survey relating to instrument use.

At the end of the 12-month review period, 10 remaining users of the system were surveyed, with a response rate of 80%. Two of the initial users were no longer in their posts. Feedback from professionals using the system noted the instrument as both relevant and user-friendly. A majority of respondents were very satisfied or extremely satisfied with the relevance of the instrument to day-to-day practice (75%) and the relevance of risk factors included (75%). Responses for ease of use of the instrument (75%) were similar. Sixty-two percent of respondents were very satisfied or extremely satisfied with the usefulness of the instrument. See Figure 2 for a graphical representation of survey responses. The mean time required for completing the instrument was reported to be 6 minutes.

## Discussion

We have described the process involved in developing a web-based violence risk-monitoring instrument for forensic community patients with psychoses. The instrument incorporates both a novel risk assessment tool and an innovative way of collecting, recording, and sharing the recorded data.

Based on a recent systematic review, we identified 10 dynamic risk factors to be used in this tool, and were able to conduct a pilot in one forensic community mental health service. We recruited 68 patients over one year, and followed them up 4–5 times on average. We found that a multidisciplinary clinical team was willing to use such a system, and received positive feedback on the web interface for relevance and ease of use.

The potential strengths of this approach to risk monitoring include ease of access to updated risk-based information in a visual format with trends over time. This could be a useful adjunct when discussing risk in clinical management meetings where patients are discussed. Graphical data relating to the escalation or reduction of risk factors could potentially be used when making decisions about recall to hospital, or justifying risk judgments when patient hospital stay is being reviewed, including for detained patients. The electronic questionnaire is also a useful reminder to clinicians about key areas to inquire about in violence risk assessment, and may help improve the quality and reliability of the documentation of these factors.

The 10 factors we identified have the benefit of being based on a recent comprehensive review of the evidence (Witt, van Dorn, & Fazel, 2013), and it is notable that four of the items used are not included in current widely used instruments, such as the HCR-20. In addition, whereas such instruments are time-consuming and require training for staff to complete, our tool was considerably simpler, quicker, and did not require specific training. This is in keeping with the need for more scalable approaches to risk assessment (Fazel, 2013).

This paper is limited to a descriptive account relating to the development of the instrument and a preliminary testing of feasibility. While our study shows some preliminary evidence of user satisfaction and utility (albeit in a small sample), this study was not able to draw conclusions about efficacy, due to sample size and length of follow-up, which would mean examining associations between increasing scores and adverse outcomes, and ultimately a reduction in adverse outcomes over time through the use of an instrument. Future work will be needed to evaluate interrater reliability, internal, and external validity, as user satisfaction, although important, is insufficient on its own as a basis for adoption by practitioners. Any future study investigating efficacy or predictive validity for outcomes such as recall to hospital or future violence would need a substantially larger sample size and follow-up period, given the relatively infrequent nature of these outcomes in a supervised community population (Coid, Hickey, Kahtan, Zhang, & Yang, 2007). Future studies would also need information from a larger number of clinicians.

Violent incidents are more frequent (Bowers et al., 2011) in an inpatient forensic mental health setting, and on this basis, we are developing a similar specific instrument for inpatients.

In summary, we report the development of a novel web-based risk monitoring instrument, and provide some preliminary evidence that it is feasible and user-friendly. Future work will determine whether it has predictive validity and whether it can be used in other settings and patient groups.

## Acknowledgments

The authors are grateful to the OXTEXT program at the University Department of Psychiatry and the Oxford National Institute for Health Research Collaboration for Leadership in Applied Health Research and Care (NIHR CLAHRC) for advice, support, and technical assistance.

The authors would also like to thank Ms. Jean Gray, Ms. Beth Morphy, Mr. Reuben Ogwo, Mrs. Jackie Newell, Mrs. Kate Helsby, and Ms. Esther Guard from the Oxfordshire Forensic Community Mental Health Team, Ms. Lisa Nicholson and Mr. Rob Evans from the Buckinghamshire Forensic Community Mental Health Team, and Mr. Fomayi Saliki and Mr. Peter Estakhrian from the Berkshire Forensic Community Mental Health Team for assistance in filling in questionnaires, Dr. Katrina Witt from the University Department of Psychiatry for assistance with developing the tool, and Hannah McMahon from the University Department of Psychiatry for technical assistance.

## Funding

Seena Fazel is funded as part of a Senior Research Fellowship in Clinical Science from the Wellcome Trust (095806) and John Geddes as part of a Senior Investigator award from the National Institute for Health Research (NIHR). This paper presents independent research partly funded by the NIHR Collaboration for Leadership in Applied Health Research and Care Oxford and NIHR PfGAR. The views expressed are those of the authors and not necessarily those of the NHS, the NIHR or the Department of Health.
